# Serotonin-Related Functional Genetic Variants Affect the Occurrence of Psychiatric and Motor Adverse Events of Dopaminergic Treatment in Parkinson’s Disease: A Retrospective Cohort Study

**DOI:** 10.3390/jpm12020266

**Published:** 2022-02-11

**Authors:** Sara Redenšek, Tanja Blagus, Maja Trošt, Vita Dolžan

**Affiliations:** 1 Pharmacogenetics Laboratory, Institute of Biochemistry and Molecular Genetics, Faculty of Medicine, University of Ljubljana, 1000 Ljubljana, Slovenia; sara.redensek@mf.uni-lj.si (S.R.); tanja.blagus@mf.uni-lj.si (T.B.); 2 Department of Neurology, University Medical Centre Ljubljana, 1000 Ljubljana, Slovenia; maja.trost@kclj.si

**Keywords:** Parkinson’s disease, motor complications, visual hallucinations, impulse control disorders, dyskinesia, polymorphism, serotonin, adverse event, pharmacogenomics

## Abstract

The serotonergic system is important in Parkinson’s disease (PD) pathogenesis as it can take over dopamine production after a large portion of dopaminergic neurons is lost through neurodegeneration. The aim of this study was to evaluate the effect of genetic variability of serotonergic genes on the occurrence of motor complications and psychiatric adverse events (AE) due to dopaminergic treatment. We enrolled 231 patients and their clinical data were collected. Genotyping was performed for eight genetic variants. Logistic regression was used for analysis. Carriers of the *HTR1A* rs6295 GC genotype (OR = 2.58; 95% CI = 1.15–5.78; *p* = 0.021), *TPH2* rs4290270 AA genotype (OR = 2.78; 95% CI = 1.08–7.03; *p* = 0.034), and at least one *TPH2* rs4570625 T allele (OR = 1.86; 95% CI = 1.00–3.44; *p* = 0.047) had increased risk for visual hallucinations (VH). Additionally, carriers of at least one *SLC6A4* *5-HTTPLR* rs25531 S (OR = 0.52; 95% CI = 0.28–0.96; *p* = 0.037) or at least one L_G_ allele (OR = 0.37; 95% CI = 0.14–0.97; *p* = 0.044) had a decreased chance for VH. Constructed haplotypes of the *TPH2* showed increased risk for VH (OR = 1.94; 95% CI = 1.06–3.55; *p* = 0.032) and impulse control disorders (OR = 5.20; 95% CI = 1.86–14.50; *p* = 0.002). Finally, individual gene–gene interactions showed decreased odds for the development of motor AE. Our findings suggest that the serotonergic pathway may play an important role in the development of AE resulting from dopaminergic treatment.

## 1. Introduction

Serotonin is a monoamine with an integral role in the human body not only as a neurotransmitter in the central nervous system, but also as a signaling molecule in the periphery [1]. It is important in many different aspects of brain activity, such as mood, memory, emotion, sleep, appetite, and motor activity [2]. Serotonin has a crucial role in the periphery as well, especially in the gastrointestinal system as 90% of total serotonin in the body is produced in the enterochromaffin cells, where it controls intestinal movement [3]. Even though it is widely accepted that degeneration of dopaminergic neurons projecting from substantia nigra pars compacta to the striatum is the main pathological hallmark of Parkinson’s disease (PD), the involvement of serotonergic neurons in PD pathology is becoming increasingly acknowledged [4].

Serotonergic neurotransmission is widely distributed in the brain and is mediated by serotonergic neurons in the raphe nuclei [5]. These neurons project to the striatum, which possesses all the machinery of the serotonergic pathway [2], meaning that serotonergic and dopaminergic pathways overlap greatly [6]. Alpha-synuclein, the misfolded protein deposited in the brain of PD patients, can be found not only in dopaminergic neurons, but in serotonergic neurons of raphe nuclei as well. Because of these intracellular proteinatious aggregates, serotonergic neurons deteriorate, although at a slower pace than dopaminergic neurons [7]. Consequently, all the components of the serotonergic pathway (e.g., serotonin transporter (SERT), tryptophan hydroxylase, serotonin, and its metabolites) become depleted [8]. However, a study using positron emission tomography (PET) with iodine-123, which determined the density of SERT, showed no difference between early-stage PD patients and controls [9]. Nevertheless, SERT binding and density was reduced in several brain regions in the advanced stages of the disease when compared to early-stage PD [10,11]. Serotonin levels were shown to be reduced as well [8].

**Figure 1:**
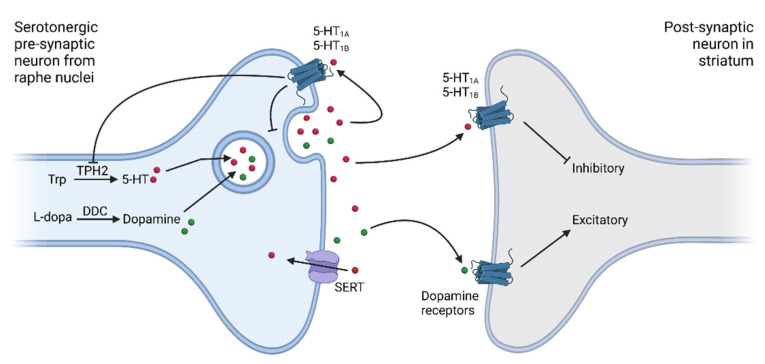
Synapse of the serotonergic pathway in the context of Parkinson’s disease pathogenesis. Dopaminergic neurons projecting from the substantia nigra to the striatum are degenerated in the process of Parkinson’s disease pathogenesis. Hence, serotonergic neurons originating in raphe nuclei take over the conversion of levodopa to dopamine. Serotonin is produced in these neurons as well. Both neurotransmitters are packaged into vesicles and released into the synaptic cleft via endocytosis. Both serotonin and dopamine then act on the postsynaptic neurons in the striatum. Serotonin has an inhibitory effect while dopamine acts in an excitatory manner. Serotonin is re-uptaken into the pre-synaptic neuron via the serotonin transporter. Simultaneously, the release of serotonin is also slowed down via the pre-synaptic serotonin receptors. Serotonergic pre-synaptic neurons do not possess the autoregulatory machinery to fine-tune the dopamine release. This means that the inhibiting feedback loop is lacking and dopamine is released from serotonergic neurons in an uncontrolled and unregulated manner.

The most effective drug for PD treatment is levodopa, which is converted to dopamine via the dopa decarboxylase (DDC) enzyme [12]. Predominantly, this conversion takes place within dopaminergic neurons [13]. However, with the increasing degeneration of dopaminergic neurons, the serotonergic neurons take over [13]. Consequently, dopamine is released from the serotonergic neurons as a false neurotransmitter together with serotonin [2,8]. Serotonin concentration in the synaptic cleft is thus decreased at the expense of dopamine [8]. Serotonin then binds to the post-synaptic serotonergic receptors where it activates the inhibitory signaling cascade. It also binds to the pre-synaptic serotonergic autoregulatory receptors, such as 5-HT_1A/1B_, to regulate the serotonin release, or is taken up by the pre-synaptic neurons via SERT [14]. The molecular circuitry of the serotonergic pathway in the context of PD is presented in Figure 1 [15,16].

Trp—tryptophan; TPH2—tryptophan hydroxylase 2; 5-HT—serotonin; L-dopa—levodopa; DDC—dopa decarboxylase; 5-HT_1A_—serotonin receptor 1A; 5-HT_1B_—serotonin receptor 1B; SERT—serotonin transporter; red dots—serotonin; green dots—dopamine. Created with BioRender.com.

Some motor symptoms of PD correlate with the serotonergic activity, for example tremors. PET imaging showed that binding of the radioligand ^11^C-WAY100635, a selective marker of 5-HT_1A_ receptors, in raphe nuclei was reduced by 27% in PD patients compared to healthy controls. There was also a significant correlation between reductions in midbrain raphe 5-HT_1A_ binding and the severity of tremors [7]. Several non-motor symptoms of PD are associated with depleted serotonin levels as well, such as depression, weight loss, fatigue, sleep disturbances, visual hallucinations (VH), psychosis, and obsessive-compulsive disorder [4,7,17,18]. Serotonin receptor agonism was already suggested as a treatment option for some non-motor manifestations of PD [4]. Additionally, restoring serotonin levels in the synaptic cleft could be a valid treatment option for parkinsonian tremors [19].

Serotonergic neurons lack the autoregulatory feedback inhibition of dopamine release (as seen in Figure 1). Therefore, levodopa treatment may lead to increased non-physiological release of dopamine from serotonergic terminals. The resulting excessive swings of striatal dopamine levels could promote levodopa induced motor complications, namely dyskinesia and motor fluctuations [7,13,20]. Consequently, selective serotonin receptor agonists, such as 5-HT_1A/1B_, were proposed to be used as anti-dyskinetic agents in PD. The proposed anti-dyskinetic mechanism of serotonin agonists is the reduction of dopamine release after levodopa administration by dampening serotonergic neuronal firing, thus attenuating peak-dose levodopa-induced dyskinesia [21,22]. In addition to dyskinesia, motor fluctuations (MF) are a very common adverse event (AE) of levodopa treatment. They usually occur after a few years of levodopa administration. On the other hand, non-motor AE, especially the psychiatric ones, such as VH and impulse control disorders (ICD), mainly occur early after initiation of treatment with dopamine agonists (DA) [23].

The serotonergic pathway evidently plays an important role in PD pathogenesis, progression, and drug response. However, the effects of genetic variants in the serotonergic pathway on drug response have scarcely been studied. Only three studies so far reported the association of the *HTR2A* rs6313 with occurrence of ICD as an adverse event of dopaminergic treatment [18,24,25], similarly as *TPH2* rs6582078 [26]. Additionally, 5-HTTLPR polymorphism was already associated with PD risk [27].

The aim of this study was to investigate the influence of genetic variability in the serotonergic pathway on the occurrence of psychiatric AE and motor complications due to dopaminergic treatment in PD. We analysed common functional genetic variants of key serotonergic genes, such as *HTR1A, HTR1B, TPH2*, and *SLC6A4.* The key rationale was to pinpoint the susceptible individuals based on the genetic fingerprint of the serotonergic pathway that would possibly benefit from the adjuvant therapy targeting this pathway.

## 2. Materials and Methods

### 2.1. Participants and Clinical Data

A cohort of 231 PD patients was enrolled in this retrospective cohort study [28]. Patients were recruited from the Department of Neurology, University Medical Centre Ljubljana, Slovenia, between October 2016 and April 2018. Inclusion criteria are listed elsewhere [28].

Patients and their caregivers underwent a structured interview to obtain demographic and clinical data of interest. Additional clinical data were obtained from medical records as well. Data on gender, body-side of disease initiation, presence of tremor, treatment with DA, age at diagnosis, disease duration, levodopa treatment duration, and levodopa equivalent dose (LED) were recorded. The focus of data collection was to gather information about the occurrence of psychiatric AE, namely ICD and VH, and motor AE, namely MF and dyskinesia. The appearence of the AE throughout the course of dopaminergic treatment was recorded as the end-point of interest.

The study protocol was approved by the Slovenian Ethics Committee for Research in Medicine (KME 42/05/16). All subjects gave written informed consent in accordance with the Declaration of Helsinki.

### 2.2. Single Nucleotide Polymorphism (SNP) Selection

We selected seven single nucleotide polymorphisms (SNPs) and one tandem repeat polymorphism in genes with a pivotal function in the serotonergic pathway, namely *HTR1A, HTR1B, TPH2*, and *SLC6A4*. We selected functional genetic variants with proven or predicted effects on transcription, translation or protein function with the minor allele frequency (MAF) above 10%. Data on the variants’ function were obtained from the available literature [29,30] or with the use of the SNP function prediction tool [31]. Genetic variants were also selected based on their previously reported associations with PD susceptibility, the investigated phenotypes, and with phenotypes similar to those. Genetic variants investigated in this study are presented in Table 1.

### 2.3. DNA Isolation and Genotyping

Peripheral blood samples were obtained for DNA extraction and genomic DNA was isolated using the FlexiGene DNA Kit (Qiagen, Hilden, Germany) in the course of our previous study [28]. Six SNPs (*HTR1A* rs6295, *HTR1B* rs13212041, *TPH2* rs1843809, *TPH2* rs7305115, *TPH2* rs4290270, *TPH2* rs4570625) were genotyped with KASPar assays (KBiosciences, Herts, UK and LGC Genomics, UK) according to manufacturer’s instructions. The cycling conditions were as follows: stage 1–94 °C, 15 min, 1 cycle; stage 2–94 °C, 20 s; 61 °C (61 °C decreasing 0.6 °C per cycle to achieve a final annealing/extension temperature of 55 °C), 60 s; 10 cycles; stage 3–94 °C, 20 s; 55 °C, 60 s; 26 cycles. To add more cycles we used the following recycling protocol: stage 1–94 °C, 20 s; 57 °C, 60 s; 3 cycles.

*SLC6A4* genotype was determined as previously described in [32]. The 5-HTTLPR L and S alleles were determined by the polymerase chain reaction (PCR) using the forward 5′-GGCGTTGCCGCTCTGAATGC-3′ and reverse 5′-GAGGGACTGAGCTGGACAACCAC-3′ oligonucleotides. Parts of the PCR reaction products were separated on 2% agarose gels to identify the L specific 528 bp fragment and the S specific 486 bp fragment. To distinguish between the L_G_ and L_A_ genotypes (*5-HTTPLR* rs25531) the rest of the PCR products were digested with the restriction enzyme HpaII overnight at 37 °C. PCR products were separated on 2% agarose gels again, providing the following bands: L_G_ = 188 bp + 151 bp + 62 bp + 127 bp; L_A_ = 339 bp + 61 bp + 127 bp; S = 297 bp + 62 bp + 127 bp. For the purpose of statistical analysis of this triallelic polymorphism we grouped genotypes according to the level of transporter’s activity/function [27] as follows: L_A_L_A_ (high activity); L_A_S + L_A_L_G_ (medium activity); and SS + SL_G_ + L_G_L_G_ (low activity).

In total, 10% of samples were genotyped in duplicate as quality control and all the results were concordant.

### 2.4. Statistical Analysis

Median and the 25th to 75th percentile range were used to describe central tendency and variability of continuous variables, while frequencies were used to describe the distribution of categorical variables. The agreement of genotype frequencies with Hardy–Weinberg equilibrium was examined by a chi-squared test. Logistic regression was used to calculate odds ratios (OR) and 95% confidence intervals (95%CI) in order to examine the associations of selected SNPs and clinical data with the risk for AE. Dominant, recessive, and additive genetic models were used for analysis according to the genotype frequencies. 

Haplotype analysis was carried out to assess the combined effect of multiple SNPs in the same gene, namely *TPH2*. On the basis of genotype data, haplotypes were constructed and analyzed using the Thesias program [33]. Only haplotypes with frequencies above 5% were included in the analysis. The most frequent haplotype was used as reference.

Gene–gene interactions were examined with logistic regression analysis. The model included two polymorphisms and their interactive term to calculate the OR, 95%CI and *p* value for each gene–gene interaction.

All statistical tests were two-sided. Bonferroni correction was used to account for multiple comparisons to prevent false positive results. Associations with *p* values up to 0.006 (0.05/8) were considered statistically significant, while *p* values between 0.006 and 0.050 were considered nominally significant. For an allelic variant with minor allele frequency of 0.32 and with 34% prevalence of an AE, this study had 80% or more power to detect OR of 0.50 or less and OR of 1.83 or more. All statistical analyses were carried out by IBM SPSS Statistics, version 21.0 (IBM Corporation, Armonk, NY, USA).

## 3. Results

### 3.1. Patients’ Characteristics

Characteristics of the patient cohort included in this study were already presented and described in a previous publication by our group [28]. They are presented in Table S1. There were 132 men and 99 women included in the study cohort. Patients’ median age at enrolment was 72.5 years (65.7–78.0). Their median age at diagnosis was 62.1 years (54.8–71.7) and their median disease duration was 7.6 years (3.8–13.6). The median dopaminergic treatment duration was 7.3 years (3.6–13.5). The frequencies of psychiatric and motor AE are presented in Table S1 as well [28].

### 3.2. SNP Genotyping Analysis

In total, six SNPs were analysed along with the triallelic genetic variant of *SLC6A4.* Data on genotype frequencies of these variants along with their locations, minor allele frequencies, and predicted functions are presented in Table 1. None of the genotype distributions deviated from the Hardy–Weinberg equlibrium as seen in Table 1.

### 3.3. Influence of Genetic Variability on the Risk for Psychiatric and Motor Adverse Events

Univariate analysis showed that carriers of the *HTR1A* rs6295 GC genotype had increased odds for development of VH (OR = 2.28; 95% CI = 1.04–5.00; *p* = 0.039). Additionally, carriers of at least one *HTR1A* rs6295 C allele showed a non-significant trend towards increased odds for development of VH (OR = 1.91; 95% CI = 0.90–4.08; *p* = 0.094). Carriers of the *TPH2* rs4290270 AA genotype (OR = 2.71; 95% CI = 1.09–6.75; *p* = 0.032) and carriers of at least one *TPH2* rs4570625 T allele (OR = 1.89; 95% CI = 1.03–3.45; *p* = 0.040) had increased odds for development of VH. Genetic variability of the serotonin transporter *SLC6A4* showed a protective role against VH. Carriers of at least one *5-HTTLPR* S variant (OR = 0.55; 95% CI = 0.30–1.00; *p* = 0.051) and patients with SS, SL_G_ or L_G_L_G_ genotypes (OR = 0.40; 95% CI = 0.16–1.03; *p* = 0.057) had lowered odds for development of VH. The results for all the investigated polymorphisms are presented in Table S2.

With regard to the occurrence of ICD, nominally significantly higher odds were observed in carriers of the *TPH2* rs4570625 GT genotype (OR = 3.03; 95% CI = 1.36–6.74; *p* = 0.007) and in carriers of at least one T allele (OR = 3.00; 95% CI = 1.37–6.57; *p* = 0.006). All data are presented in the Table S3.

With regard to motor complications, no significant associations between the investigated genetic variants and MF and dyskinesia were detected in the univariate analysis as presented in Tables S4 and S5, respectively.

The associations described above were adjusted for (1) age at diagnosis for VH and (2) age at diagnosis and treatment with DA for ICD as we have previously reported that these clinical parameters were significantly associated with assessed AE [28]. All of the associations observed in univariate analysis remained nominally significant after adjustment. In addition, the association between VH and the genotype of *SLC6A4* became nominally significant. The adjusted associations are presented in Table 2.

### 3.4. Influence of Haplotypes on the Risk for Psychiatric and Motor Adverse Events

Constructed *TPH2* haplotypes were analysed in relation to the occurrence of AE. There were five distinct haplotypes included in the analysis with the frequency above 5%. The considered haplotypes covered more than 90% of genetic variability. The TGTG haplotype was carried by more than 50% of patients and was thus used as a reference. The TAAT haplotype increased the odds for the development of VH (OR = 1.94; 95% CI = 1.06–3.55; *p* = 0.032). The TATT haplotype increased the odds for the development of ICD (OR = 5.20; 95% CI = 1.86–14.50; *p* = 0.002). The associations of the most common *TPH2* haplotypes with AE are presented in Table 3.

### 3.5. Influence of Gene–Gene Interactions on the Risk for Psychiatric and Motor Adverse Events

We tested the following gene pairs in our interaction analysis: *TPH2-SLC6A4, TPH2-HTR1A, TPH2-HTR1B, SLC6A4-HTR1A,* and *SLC6A4-HTR1B.* We observed that interactions between the analysed genes mainly affect the occurrence of motor complications. Carriers of at least one *TPH2* rs4290270 A allele and at least one *5-HTTLPR* S variant had lower odds for development of dyskinesia (OR = 0.29; 95% CI = 0.09–0.91; *p* = 0.034). Furthermore, carriers of at least one *TPH2* rs7305115 A allele and at least one *HTR1A* rs6295 C allele had lower odds for MF (OR = 0.20; 95% CI = 0.06–0.71; *p* = 0.013) as well as dyskinesia (OR = 0.16; 95% CI = 0.04–0.60; *p* = 0.006). Finally, carriers of at least one *TPH2* rs4570625 T allele and at least one *HTR1A* rs6295 C allele had lower chance of developing MF (OR = 0.28; 95% CI = 0.08–0.95; *p* = 0.041) as well. Significant associations are presented in Table 4, but all the results are presented in Table S6.

## 4. Discussion

In the present study we tested the associations between seven common functional genetic variants in four serotonergic pathway genes and the AE related to dopaminergic treatment of PD. We observed important associations of assessed genetic variants with the occurrence of VH and ICD, which are the most common psychiatric AE of dopaminergic treatment. These associations were consistently observed in univariate analysis, after adjustment for clinical parameters, and in haplotype analysis. However, associations of the studied genetic variants with the occurrence of motor complications after levodopa treatment were observed only in the gene–gene interaction analysis.

The *HTR1A* rs6295 GC genotype was associated with increased risk for VH. Additionally, the dominant model showed a slight trend towards increased risk for VH in carriers of at least one C allele. This SNP affects the transcription factor binding site, with the effect dependent on the type of neurons. Upon binding to the wild-type G allele sequence transcription factor Deaf1 lowers transcription of the autoreceptor 5-HT_1A_ in serotonergic neurons, while it increases transcription of the post-synaptic 5-HT_1A_ receptor in the non-serotonergic neurons [34]. The G > C substitution in the polymorphic rs6295 allele prevents binding of this transcription factor, which would mean that the 5-HT_1A_ expression is on the contrary increased in serotonergic neurons and decreased in the non-serotonergic neurons [35,36]. Consequently, release of serotonin from the pre-synaptic neurons might be decreased leading to possibly dampened inhibitory serotonin signaling in the post-synaptic neurons. This might lead to enhanced dopamine signaling in the post-synaptic neurons, which could result in a higher chance for VH development [35,36]. This hypothetic mechanism would need further studies to be confirmed.

The *TPH2* rs4290270 AA genotype and the *TPH2* rs4570625 T allele were associated with increased odds for VH development as well. These two SNPs have not been investigated in connection to PD-related phenotypes before. Tryptophan hydroxylase 2 is mainly expressed in serotonergic neurons and is the rate-limiting step in the synthesis of serotonin [37]. The investigated SNPs may affect splicing and transcription factor binding, respectively [31]. As a consequence, these two SNPs might increase the odds for VH development. However, the pathway leading to that can only be speculated at this point.

We observed a slight trend towards an association between the *SLC6A4* genotype and the occurrence of VH, which became nominally significant after the adjustment for clinical parameters. The *SLC6A4* polymorphic variants decrease the transcription factor binding and the transporter’s transcriptional activity, which can lead to reduced serotonin re-uptake to the pre-synaptic neuron [32,38]. Thus, the concentration of serotonin in the synaptic cleft is increased, which might lead to a lower dopaminergic tone possibly resulting in a lower risk for VH. Additionally, due to high serotonin concentration in the synaptic cleft the release of dopamine from pre-synaptic serotonergic neurons might be decreased, which could add to the low dopaminergic signal transduction. Whether this speculated mechanism holds true should be further experimentally confirmed.

It has been described several times how the function of serotonin system is important in the development of VH in different pathologies, such as schizophrenia [39], dementia [40], and PB [41]. Importantly, pimavanserin, a 5-HT_2A_ inverse agonist, was approved for treatment of psychosis including VH in PD patients [42,43], indicating the important role of the serotonergic system in the development of VH. The exact pathophysiology is not known yet. However, there is speculation that VH can contribute to the imbalance in dopamine and serotonin neurotransmission in mesencephalic striatal and extrastriatal projections [42].

We have also observed an association between the *TPH2* rs4570625 and increased odds for the development of ICD. The polymorphic allele of this SNP is associated with reduced serotonin levels due to lowered synthesis [44]. Reduction in serotonin activity/transmission might diminish tonic inhibition of midbrain dopaminergic neurons and lead to impulsive behaviour due to increased dopamine release [26]. Speculations exist that the degeneration of serotonergic neurons leading to reduced serotonin production contributes to ICD development and that selective serotonin re-uptake inhibitors, or perhaps direct serotonergic agonists targeting specific receptor subtypes, could alleviate ICD [45].

We did not observe any significant associations between the investigated genetic variants and motor complications in the univariate analysis. This was slightly unexpected since MF and dyskinesia partly occur due to the fact that levodopa becomes converted to dopamine in serotonergic neurons, which lack the dopamine autoregulatory machinery, after a large proportion of dopaminergic neurons have degenerated [7]. Maybe the crude serotonin-related individual genetic factors did not have an effect strong enough to show significant associations on their own, especially in a rather small cohort of patients. Additionally, maybe the function of dopaminergic pre-synaptic neurons in the patient cohort was on average adequate to adjust for the defects in the serotonergic neurons. It is possible that we would be able to detect some differences between the groups of genotypes later on when more dopaminergic neurons would be deteriorated.

A haplotype analysis revealed similar results as the univariate and multivariate analyses. The *TPH2* TAAT haplotype carriers had almost two-fold increased chance for development of VH in comparison to the carriers of the reference haplotype. If we compare these results to the univarate analysis, we can see that in both scenarios the rs4290270 A allele and the rs4570625 T allele were nominally significantly associated with the occurrence of VH. Additionally, the TATT haplotype carriers had more than five-fold higher odds for development of ICD. If we again compare that to the univariate analysis, we can conclude that the rs4570625 T allele is of high importance for the development of ICD. This SNP was already associated with different psychiatric disorders, such as depression [46,47,48], poor inhibitory processing [44], and aggressive traits [49].

Interestingly, the gene–gene interaction analysis showed nominally significant associations with the occurrence of motor complications, both MF and dyskinesia. The interaction between *TPH2* rs7305115 and *5-HTTLPR* showed a protective role in dyskinesia development. These genetic variants might affect serotonin synthesis and decrease serotonin re-uptake [30], respectively. Consequently, less serotonin might be released into the synaptic cleft. Decreased serotonin re-uptake might also lead to less dopamine being released as a false neurotransmitter due to a smaller stimulus. Further interactions can be explained by a similar mechanism. The interactions between the *TPH2* SNPs and the *HTR1A* rs6295 polymorphic alleles overall may lead to decreased dopamine release from the pre-synaptic serotonergic neurons, which could protect against non-physiological concentrations and spikes of dopamine in the synaptic cleft. By contrast to VH, where the *HTR1A* rs6295 polymorphic allele increased the chances for their occurrence, the motor complications occur much later in disease progression, when pre-synaptic dopaminergic neurons are almost completely degenerated and dopamine synthesis is probably almost completely dependent on the serotonergic neurons.

Our study presents important novel findings; however, some limitations should be borne in mind when interpreting the results. A study cohort is of moderate size, but still comparable to other pharmacogenetic studies performed in the cohorts of PD patients and of a uniform genetic background as well. Additionally, the end-points were only considered as binary categorical variables since clinical scales are not routinely used to assess AE in PD. It is possible that clinical scale scores would give us an opportunity to analyse associations in more depth. Our study was also limited to the investigation of genetic variability of the serotonergic pathway in regard to the pre-synaptic serotonergic neurons and we did not include any of the post-synaptic signaling mediators, hence we did not evaluate *HTR2A*. Additionally, our results were not validated in an independent cohort of PD patients. There is a chance we have missed some cases of AE simply due to the fact that they might have occurred after the patients had been included in the study. Also, a prospective study could give us a better insight into the predictive capacity of the candidate genetic variants.

Nevertheless, the presented study showed some interesting findings that have not been pointed out before. The less functional allele of the *5-HTTLPR* protects against VH and later in the disease course also against dyskinesia. This indicates that selective serotonin re-uptake inhibitors could possibily be used to treat or prevent these AE [8,50,51]. Additionally, *HTR1A* rs6295 affected the occurrence of VH and motor complications, but in an opposite manner. This could be explained by the fact that these respective AE occur at different stages of the disease—very early or rather late, respectively. Thus, HTR1A agonists could be used to prevent or treat these adverse events as well [8,51,52]. Additionally, the genetic variants pointed out could also serve as predictive biomarkers of the analysed AE and help guide the treatment of PD patients.

## 5. Conclusions

To the best of our knowledge, this is one of very few studies to analyse the serotonergic pathway in association with AE of dopaminergic treatment in PD, although it is evident from the literature that these two systems are highly interconnected in terms of PD pathogenesis and the development of AE as well. The significant and nominally significant associations reported support the idea that compounds acting on the serotonergic system could be beneficial in the prevention or treatment of AE due to dopaminergic treatment. Thus, these novel findings may contribute to better and more personalized care for PD patients.

## Figures and Tables

**Table 1:** Genotype frequencies and characteristics of the genetic variants studied in our Parkinson’s disease (PD) cohort.

Gene	Polymorphism	Location in Gene	MAF	Function Prediction *	Genotype	*N* (%)	HWE Equilibrium *p* Value
*HTR1A*	rs6295 c.-1019G>C	5′UTR	0.46	Influences transcription factor binding	GG	60 (26.0)	0.632
					GC	119 (51.5)	
					CC	52 (22.5)	
*HTR1B*	rs13212041 c.* 824G>A	3′UTR	0.19	Influences miRNA binding	TT	154 (66.7)	0.778
					CT	70 (30.3)	
					CC	7 (3.0)	
*TPH2*	rs1843809 c.608+5263G>T	Intron	0.15	Possibly in LD with a causative variant	TT	164 (71.0)	0.908
					GT	61 (26.4)	
					GG	6 (2.6)	
	rs7305115 g.45237A>G	Coding region	0.42	Influences splicing	GG	80 (34.6)	0.888
					AG	111 (48.1)	
					AA	40 (17.3)	
	rs4290270 p.Ala375=	Coding region	0.38	Influences splicing	TT	83 (35.9)	0.172
					AT	119 (51.5)	
					AA	29 (12.6)	
	rs4570625 c.-844G>T	5′UTR	0.21	Influences transcription factor binding	GG	133 (57.6)	0.335
					GT	88 (38.1)	
					TT	10 (4.3)	
*SLC6A4*	*5-HTTLPR*	5′UTR	0.43	Affects transcriptional efficiency [30]	LL	84 (36.4)	0.454
					LS	106 (45.9)	
					SS	41 (17.7)	
	*5-HTTLPR* rs25531 c.-1936A>G	5′UTR	0.45	Affects transcriptional activity [29] and transcription factor binding	L_A_L_A_	75 (31.2)	0.970
					L_A_S, L_A_L_G_	113 (48.9)	
					SS, SL_G_, L_G_L_G_	43 (18.6)	

MAF—minor allele frequency; HWE—Hardy-Weinberg equilibrium; LD—linkage disequilibrium. * According to the single nucleotide polymorphism (SNP) function prediction tool [31].

**Table 2:** Nominally significant associations adjusted for significant clinical parameters—multivariate analysis.

Association			Genotype	OR Adj. **	95% CI Adj.	*p* Value Adj.
Adverse Event	Adjusted for *	SNP				
Visual hallucinations	Age at diagnosis	*HTR1A* rs6295	GG	Ref.		
			GC	**2.58**	**1.15–5.78**	**0.021**
			CC	1.23	0.46–3.31	0.676
		*TPH2* rs4290270	TT	Ref.		
			AT	1.14	0.58–2.27	0.702
			AA	**2.78**	**1.08–7.03**	**0.034**
		*TPH2* rs4570625	GG	Ref.		
			GT+TT	**1.86**	**1.00–3.44**	**0.047**
		*SLC6A4 5HTTLPR*	LL	Ref.		
			LS+SS	**0.52**	**0.28–0.96**	**0.037**
		*SLC6A4* *5HTTLPR* rs25531	L_A_L_A_	Ref.		
			L_A_S, L_A_L_G_	0.54	0.28–1.05	0.069
			SS, SL_G_, L_G_L_G_	**0.37**	**0.14–0.97**	**0.044**
Impulse control disorders	Age at diagnosis Ever being treated with DA	*TPH2* rs4570625	GG	Ref.		
			GT	**3.00**	**1.27–7.07**	**0.012**
			TT	4.37	0.74–25.97	0.105
			GT+TT	**3.10**	**1.34–7.18**	**0.008**

* Results of the univariate analysis of associations between clinical parameters and adverse events are reported in [28]. ** Adjusted odds ratios (OR) present a fold change over the reference genotype. Nominally significant results are printed in bold text.

**Table 3:** Significant and nominally significant associations between *TPH2* haplotypes and adverse events.

*TPH2* Haplotype *	Frequency		Visual Hallucinations	Impulse Control Disorders	Motor Fluctuations	Dyskinesia
**TGTG**	51.6	Reference				
**TAAT**	15.6	OR	**1.94**	1.77	0.80	1.30
		95%CI	**1.06–3.55**	0.74–4.21	0.46–1.39	0.75–2.25
		*p* value	**0.032**	0.197	0.433	0.343
**GAAG**	14.0	OR	0.80	1.09	0.93	0.88
		95%CI	0.40–1.58	0.40–2.96	0.51–1.66	0.49–1.60
		*p* value	0.516	0.871	0.795	0.679
**TATT**	6.5	OR	1.20	**5.20**	1.79	1.99
		95%CI	0.45–3.21	**1.86–14.50**	0.82–3.90	0.92–4.32
		*p* value	0.720	**0.002**	0.143	0.080
**TGAG**	5.2	OR	1.27	2.24	1.35	1.51
		95%CI	0.44–3.67	0.61–8.26	0.53–3.43	0.60–3.80
		*p* value	0.652	0.227	0.533	0.382

* Alleles within the *TPH2* haplotype are presented in the following order: rs1843809, rs7305115, rs4290270, rs4570625. Statistically significant and nominally significant results are printed in bold text.

**Table 4:** Nominally significant associations in the gene–gene interaction analysis.

Interaction		Visual Hallucinations	Impulse Control Disorders	Motor Fluctuations	Dyskinesia
*TPH2* rs4290270 and *5-HTTLPR*	OR	0.98	0.78	0.63	**0.29**
	95%CI	0.27–3.58	0.17–3.57	0.20–1.93	**0.09–0.91**
	*p* value	0.972	0.743	0.416	**0.034**
*TPH2* rs7305115 and *HTR1A* rs6295	OR	/	0.15	**0.20**	**0.16**
	95%CI	/	0.02–1.59	**0.06–0.71**	**0.04–0.60**
	*p* value	/	0.116	**0.013**	**0.006**
*TPH2* rs4570625 and *HTR1A* rs6295	OR	0.26	0.13	**0.28**	0.37
	95%CI	0.04–1.54	0.01–1.30	**0.08–0.95**	0.11–1.28
	*p* value	0.137	0.082	**0.041**	0.116

Nominally significant results are printed in bold text.

## Data Availability

The data presented in this study are available on request from the corresponding author. The data are not publicly available because the study is still ongoing.

## Supplementary Materials

The following supporting information can be downloaded at: https://www.mdpi.com/article/10.3390/jpm12020266/s1, Table S1: Clinical characteristics of the patient cohort; Table S2: Visual hallucinations and their associations with assessed polymorphisms; Table S3: Impulse control disorders and their associations with assessed polymorphisms; Table S4: Motor fluctuations and their associations with assessed polymorphisms; Table S5: Dyskinesia and their associations with assessed polymorphisms; Table S6: Analysis of interactions between genetic variants and adverse events.
