# Supplementary material for: Serotonin-Related Functional Genetic Variants Affect the Occurrence of Psychiatric and Motor Adverse Events of Dopaminergic Treatment in Parkinson’s Disease: A Retrospective Cohort Study
Source: J Pers Med. 2022 Feb 11;12(2):266. doi: 10.3390/jpm12020266 (PMC8875505; doi:10.3390/jpm12020266)
Supplement: Supplementary file 1 [file jpm-12-00266-s001.zip › jpm-1538014-supplementary.pdf]

Table S1: Clinical characteristics of the patient cohort

Characteristic		All patients (N = 231)
Gender	Male (%)	132 (57.1)
	Female (%)	99 (42.9)
Side of disease initiation	Left (%)	91 (39.4)
	Both (%)	21 (9.1)
	Right (%)	119 (51.5)
Tremor-predominant PD	No (%)	46 (19.9)
	Yes (%)	185 (81.1)
Ever being treated with DAs**	No (%)	57 (25.1)
	Yes (%)	170 (74.9)
Age at diagnosis	Median (25%-75%), years	62.1 (54.8 – 71.7)
Disease duration	Median (25%-75%), years	7.6 (3.8 – 13.6)
Dopaminergic treatment duration***	Median (25%-75%), years	7.3 (3.6-13.5)
Levodopa treatment duration**	Median (25%-75%), years	6.2 (2.4 – 11.2)
LED at enrolment*,**	Median (25%-75%), mg/day	975 (600 – 1363.5)
**Adverse event**	**Number (%) of patients experiencing the adverse event**	
Motor fluctuations	123 (53.2)	
Dyskinesia	101 (43.7)	
Visual hallucinations****	57 (24.7)	
Impulse control disorders****	32 (13.9)	

*LED calculated according to Tomlinson et al.

**Data missing for four patients.

***Data missing for three patients.

****Data missing for one patient.

Table S2: Visual hallucinations and their associations with assessed polymorphisms

Gene	SNP	Genotype	Visual hallucinations		
			OR	95% CI	*p* value
HTR1A	**rs6295**	GG	Ref.		
		**GC**	**2.284**	**1.044-4.995**	**0.039**
		CC	1.190	0.452-3.133	0.724
		GC + CC	1.911	0-896-4.075	0.094
HTR1B	rs13212041	TT	Ref.		
		GT	1.393	0.739-2.623	0.305
		GG	0.000	0.000	0.999
		GT + GG	1.219	0.652-2.278	0.535
TPH2	rs1843809	TT	Ref.		
		GT	0.669	0.325-1-372	0.272
		GG	0.682	0.074-6.267	0.735
		GT + GG	0.669	0.333-1.345	0.259
TPH2	rs7305115	GG	Ref.		
		AG	1.551	0.768-3.136	0.221
		AA	2.086	0.876-4.969	0.097
		AG + AA	1.685	0.867-3.277	0.124
TPH2	**rs4290270**	TT	Ref.		
		AT	1.060	0.539-2.083	0.866
		**AA**	**2.708**	**1.087-6.745**	**0.032**
		AT + AA	1.304	0.689-2.467	0.415
TPH2	**rs4570625**	GG	Ref.		
		GT	1.805	0.967-3.368	0.064
		TT	2.718	0.715-10.337	0.142
		**GT + TT**	**1.886**	**1.031-3.452**	**0.040**
SLC6A4	***5-HTTLPR***	LL	Ref.		
		LS	0.59	0.31-1.12	0.106
		SS	0.45	0.18-1.14	0.092
		**LS+SS**	**0.55**	**0.30-1.00**	**0.051**
SLC6A4	**rs25531**	LaLa	Ref.		
		LaS, LaLg	0.57	0.30-1.09	0.090
		**SS, LgLg, SLg**	**0.40**	**0.16-1.03**	**0.057**

Table S3: Impulse control disorders and their associations with assessed polymorphisms

Gene	SNP	Genotype	Impulse control disorder		
			**OR**	**95% CI**	***p* value**
HTR1A	rs6295	GG	Ref.		
		GC	0.728	0.306-1.736	0.474
		CC	0.778	0.273-2.215	0.638
		GC + CC	0.743	0.330-1.676	0.475
HTR1B	rs13212041	TT	Ref.		
		GT	1.240	0.559-2.751	0.597
		GG	1.108	0.127-9.693	0.926
		GT + GG	1.228	0.566-2.664	0.604
TPH2	rs1843809	TT	Ref.		
		GT	0.881	0.372-2.081	0.772
		GG	0.000	0.000	0.999
		GT + GG	0.805	0.342-1.895	0.619
TPH2	rs7305115	GG	Ref.		
		AG	1.246	0.516-3.008	0.625
		AA	1.972	0.697-5.579	0.200
		AG + AA	1.429	0.627-3.255	0.396
TPH2	rs4290270	TT	Ref.		
		AT	0.502	0.216-1.169	0.110
		AA	1.643	0.586-4.604	0.345
		AT + AA	0.688	0.323-1.466	0.332
TPH2	**rs4570625**	GG	Ref.		
		**GT**	**3.029**	**1.362-6.736**	**0.007**
		TT	2.750	0.519-14.578	0.235
		**GT + TT**	**3.000**	**1.370-6.567**	**0.006**
SLC6A4	*5-HTTLPR*	LL	Ref.		
		LS	0.70	0.32-1.55	0.378
		SS	0.37	0.10-1.37	0.138
		LS+SS	0.61	0.29-1.29	0.193
SLC6A4	rs25531	LaLa	Ref.		
		LaS, LaLg	0.96	0.42-2.20	0.923
		SS, LgLg, SLg	0.79	0.25-2.44	0.677

Table S4: Motor fluctuations and their associations with assessed polymorphisms

Gene	SNP	Genotype	Motor fluctuations		
			OR	95% CI	*p* value
HTR1A	rs6295	GG	Ref.		
		GC	1.019	0.546-1.902	0.953
		CC	0.701	0.333-1.478	0.351
		GC + CC	0.909	0.504-1.641	0.752
HTR1B	rs13212041	TT	Ref.		
		GT	0.930	0.528-1.637	0.801
		GG	2.195	0.413-11.662	0.356
		GT + GG	1.000	0.578-1.730	1.000
TPH2	rs1843809	TT	Ref.		
		GT	0.816	0.453-1.469	0.497
		GG	1.685	0.300-9.459	0.553
		GT + GG	0.868	0.491-1.534	0.626
TPH2	rs7305115	GG	Ref.		
		AG	0.588	0.328-1.052	0.073
		AA	0.902	0.418-1-948	0.793
		AG + AA	0.658	0.380-1.140	0.135
TPH2	rs4290270	TT	Ref.		
		AT	0.607	0.344-1.071	0.085
		AA	0.812	0.346-1.908	0.633
		AT + AA	0.642	0.372-1.108	0.112
TPH2	rs4570625	GG	Ref.		
		GT	1.131	0.658-1.942	0.656
		TT	0.600	0.162-2.224	0.445
		GT + TT	1.060	0.628-1.789	0.827
SLC6A4	*5-HTTLPR*	LL	Ref.		
		LS	1.41	0.79-2.51	0.244
		SS	0.86	0.41-1.83	0.701
		LS+SS	1.23	0.72-2.10	0.455
SLC6A4	rs25531	LaLa	Ref.		
		LaS, LaLg	1.44	0.80-2.60	0.222
		SS, LgLg, SLg	0.89	0.42-1.89	0.768

Table S5: Dyskinesia and their associations with assessed polymorphisms

Gene	SNP	Genotype	Dyskinesia		
			OR	95% CI	*p* value
HTR1A	rs6295	GG	Ref.		
		GC	0.858	0.461-1.599	0.631
		CC	0.615	0.288-1.314	0.210
		GC + CC	0.777	0.431-1.403	0.403
HTR1B	rs13212041	TT	Ref.		
		GT	1.221	0.692-2.155	0.490
		GG	1.027	0.222-4.746	0.973
		GT + GG	1.202	0.694-2.084	0.512
TPH2	rs1843809	TT	Ref.		
		GT	0.845	0.465-1.532	0.578
		GG	0.608	0.108-3.413	0.572
		GT + GG	0.821	0.461-1.462	0.503
TPH2	rs7305115	GG	Ref.		
		AG	0.865	0.484-1.545	0.624
		AA	1.106	0.517-2.367	0.796
		AG + AA	0.924	0.535-1.594	0.776
TPH2	rs4290270	TT	Ref.		
		AT	0.736	0.418-1.298	0.290
		AA	1.209	0.519-2.818	0.661
		AT + AA	0.813	0.474-1.397	0.454
TPH2	rs4570625	GG	Ref.		
		GT	1.683	0.976-2.901	0.061
		TT	1.608	0.444-5.829	0.470
		GT + TT	1.675	0.988-2.840	0.056
SLC6A4	*5-HTTLPR*	LL	Ref.		
		LS	1.36	0.76-2.43	0.294
		SS	0.94	0.44-2.02	0.876
		LS+SS	1.23	0.72-2.12	0.452
SLC6A4	rs25531	LaLa	Ref.		
		LaS, LaLg	1.19	0.66-2.14	0.568
		SS, LgLg, SLg	0.80	0.37-1.72	0.562

Table S6: Analysis of interactions between genetic variants and adverse events

Interaction		Visual hallucinations	Impulse control disorders	Motor fluctuations	Dyskinesia
TPH2 rs1843809 *SLC6A4 5-HTTLPR*	OR	1.01	1.63	1.39	0.56
	95%CI	0.24-4.16	0.28-9.48	0.42-4.60	0.17-1.89
	p value	0.993	0.587	0.591	0.351
TPH2 rs7305115 *SLC6A4 5-HTTLPR*	OR	1.87	1.44	0.82	0.37
	95%CI	0.48-7.35	0.27-7.62	0.27-2.56	0.12-1.16
	p value	0.368	0.668	0.736	0.088
TPH2 rs4290270 *SLC6A4 5-HTTLPR*	OR	0.98	0.78	0.63	**0.29**
	95%CI	0.27-3.58	0.17-3.57	0.20-1.93	**0.09-0.91**
	p value	0.972	0.743	0.416	**0.034**
TPH2 rs4570625 *SLC6A4 5-HTTLPR*	OR	2.89	1.22	0.92	0.94
	95%CI	0.82-10.18	0.25-5.99	0.31-2.76	0.31-2.86
	p value	0.100	0.803	0.887	0.919
TPH2 rs1843809 *HTR1A* rs6295	OR	1.02	4.40	1.20	0.69
	95%CI	0.16-6.44	0.41-64.51	0.32-4.49	0.18-2.59
	p value	0.980	0.219	0.789	0.577
TPH2 rs7305115 *HTR1A* rs6295	OR	/	0.15	**0.20**	**0.16**
	95%CI	/	0.02-1.59	**0.06-0.71**	**0.04-0.60**
	p value	/	0.116	**0.013**	**0.006**
TPH2 rs4290270 *HTR1A* rs6295	OR	0.42	0.75	1.17	0.87
	95%CI	0.07-2.52	0.24-3.93	0.34-4.09	0.26-2.98
	p value	0.342	0.731	0.803	0.828
TPH2 rs4570625 *HTR1A* rs6295	OR	0.26	0.13	**0.28**	0.37
	95%CI	0.04-1.54	0.01-1.30	**0.08-0.95**	0.11-1.28
	p value	0.137	0.082	**0.041**	0.116
TPH2 rs1843809 *HTR1B* rs13212041	OR	0.77	0.30	1.11	0.74
	95%CI	0.18-3.28	0.05-2.04	0.34-3.65	0.22-2.46
	p value	0.727	0.221	0.863	0.617
TPH2 rs7305115 *HTR1B* rs13212041	OR	0.45	2.88	1.25	1.19
	95%CI	0.12-1.79	0.44-18.87	0.39-4.00	0.38-3.81
	p value	0.258	0.269	0.710	0.764
TPH2 rs4290270 *HTR1B* rs13212041	OR	0.88	1.19	0.47	0.71
	95%CI	0.23-3.30	0.25-5.73	0.15-1.52	0.23-2.24
	p value	0.845	0.832	0.208	0.561

TPH2 rs4570625 *HTR1B* rs13212041	OR	0.95	2.50	1.25	2.39
	95%CI	0.27-3.36	0.46-13.65	0.41-3.80	0.77-7.49
	p value	0.937	0.291	0.696	0.134
SLC6A4 5-HTTLPR *HTR1A* rs6295	OR	0.18	0.38	0.88	0.67
	95%CI	0.03-1.09	0.07-2.12	0.25-3.03	0.19-2.34
	p value	0.062	0.267	0.833	0.524
SLC6A4 5-HTTLPR *HTR1B* rs13212041	OR	1.60	3.09	0.61	0.62
	95%CI	0.45-5.73	0.62-15.48	0.20-1.90	0.20-1.95
	p value	0.469	0.170	0.391	0.416
HTR1A rs6295 *HTR1B* rs13212041	OR	0.83	0.19	0.88	0.67
	95%CI	0.17-3.97	0.03-1.04	0.25-3.03	0.19-2.34
	p value	0.816	0.055	0.833	0.524
